# The Influence of Spousal Genotype on Alcohol Problems in Marriage

**DOI:** 10.1111/acer.70399

**Published:** 2026-08-20

**Authors:** Sally I‐Chun Kuo, Vivia V. McCutcheon, Fazil Aliev, Kathleen K. Bucholz, Danielle M. Dick, Howard J. Edenberg, Victor Hesselbrock, John R. Kramer, Dongbing Lai, Martin H. Plawecki, Marc A. Schuckit, Jessica E. Salvatore

**Affiliations:** ^1^ Department of Psychiatry, Robert Wood Johnson Medical School Rutgers University New Brunswick New Jersey USA; ^2^ Department of Psychiatry Washington University School of Medicine St. Louis Missouri USA; ^3^ Rutgers Addiction Research Center in the Brain Health Institute Piscataway New Jersey USA; ^4^ Department of Medical and Molecular Genetics Indiana University School of Medicine Indianapolis Indiana USA; ^5^ Department of Biochemistry and Molecular Biology Indiana University School of Medicine Indianapolis Indiana USA; ^6^ Department of Psychiatry University of Connecticut School of Medicine Farmington Connecticut USA; ^7^ Department of Psychiatry University of Iowa Carver College of Medicine Iowa City Iowa USA; ^8^ Center for Computational Biology and Bioinformatics Indiana University School of Medicine Indianapolis Indiana USA; ^9^ Department of Psychiatry Indiana University School of Medicine Indianapolis Indiana USA; ^10^ Department of Psychiatry University of California San Diego School of Medicine San Diego California USA

**Keywords:** alcohol use disorder, marriage, polygenic scores, social genetic effects, spousal influence

## Abstract

**Background:**

The field's conventional understanding of how genetic factors impact risk for alcohol use disorder (AUD) typically focuses on direct genetic effects, or how an individual's own genetic predispositions are associated with the likelihood of experiencing clinically significant alcohol problems. More recently, studies of behavioral health outcomes in preclinical and human studies have demonstrated the potential importance of social genetic effects or the influence of a social partner's genotype on substance use and related outcomes. In this study, we sought to characterize social genetic effects for AUD, specifically in the context of marriage.

**Methods:**

The sample included 660 opposite‐sex spousal dyads from the Collaborative Study on the Genetics of Alcoholism, restricted to individuals who were genetically similar to European reference panels. Measured and latent indicators of genetic risk included polygenic scores of problematic alcohol use (PGS_PAU_) and parental history of AUD (PH_AUD_), respectively. The outcome was Diaganostic and Statistical Manual (DSM)‐5 alcohol use disorder criterion count (AUDcrit) during marriage. Multilevel models accounted for the non‐independence of partners' data, including correlations between partners' genetic risk and residual covariance in AUDcrit.

**Results:**

After accounting for direct genetic effects (i.e., the influence of one's own genetic predispositions) and the correlations between partners' genetic predispositions, we found that having a spouse with higher PGS_PAU_ was associated with higher AUDcrit (B = 0.084, 95% CI [0.035, 0.132]). This social genetic effect was robust after adjusting for both partners' educational attainment. Spousal PH_AUD_ was not associated with AUDcrit. Exploratory analyses indicated that the social genetic effect of spousal PGS_PAU_ was stronger among male target individuals.

**Conclusions:**

Findings highlight the potential importance of social genetic effects for understanding the pathways from genotype to alcohol use disorder and the need for further investigation of latent measures of genetic predispositions in studies of social genetic effects.

## Graphical abstract

A spouse's polygenic score for problematic alcohol use was associated with the target individual's alcohol use disorder criterion count during marriage, above and beyond the effect of the target individual's own polygenic score. This pattern is consistent with a social genetic effect, or the influence of a social partner's genotype on an individual's outcome. The findings highlight marriage as an important social context for understanding pathways from genotype to alcohol use disorder.

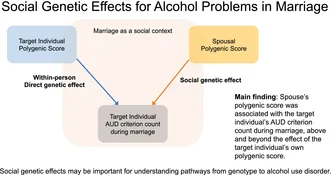

## 1 Introduction

Multiple factors influence risk for alcohol use disorder (AUD) across the lifespan, including genetic predispositions and environmental influences such as social and cultural contexts (Sher et al. 2005). Genetic epidemiological studies underscore the importance of both genetic and environmental factors, as well as their interplay, in the development of AUD (Verhulst et al. 2015; Young‐Wolff et al. 2011). Clarifying how these influences operate across development is central to the goal of building a comprehensive understanding of AUD etiology, which in turn can inform more personalized approaches to prevention and treatment (Boness and Witkiewitz 2023). There is increasing recognition of “social genetic effects,” whereby the genotypes of peers and social partners may also be associated with behavioral and mental health outcomes through their effect on behaviors of individuals with whom they interact (Baud et al. 2017; Sotoudeh et al. 2019). In this report, we test whether a spouse's genetic predispositions for AUD are associated with an individual's clinically significant alcohol problems during marriage.

Marriage is a salient interpersonal context for understanding the natural history of alcohol use and AUD (Kendler et al. 2016, 2018; Lee et al. 2015; Leonard and DAS Eiden 1999; Leonard and Eiden 2007; Leonard and Homish 2005; Rönkä et al. 2002). This reflects both the relative ubiquity of marriage (Pew Research Center 2019) and the substantial amount of leisure time that spouses spend together (Genadek et al. 2016). Consistent with the idea that social role transitions can constrain individual behavior (Yamaguchi and Kandel 1985), the transition to marriage is commonly associated with reductions in alcohol use and risk for AUD (Bachman et al. 1984; Horn et al. 2013). Although marriage typically has a protective effect on risk of AUD (Leonard and Rothbard 1999), evidence also suggests that patterns of alcohol use and AUD risk may be contagious between spouses. For example, in analyses of nearly 10,000 marital dyads from the Swedish national registers, a spouse's AUD registration during marriage was associated with a rapid and substantial increase in the risk of the other partner receiving an AUD registration in the subsequent period (hazard ratios = 13.82 in women and 9.21 in men; Kendler et al. 2018).

Evidence for spousal influence at the phenotypic (behavioral) level is also of interest from a genetic perspective, given that alcohol use itself is genetically influenced. Traditionally, research on the genetics of AUD has focused on direct genetic effects, whereby an individual's own genetic predispositions influence the likelihood of experiencing clinically significant alcohol problems through “within‐the‐skin” mechanisms such as sensitivity to alcohol (Heath et al. 1999) or traits like impulsivity and sensation seeking (Slutske et al. 2002). However, in close relationships such as marriage, a spouse's genotype is also an environment for the target individual. Consistent with this possibility, in the UK Biobank, individuals married to spouses carrying the protective A allele of the *ADH1B* SNP rs1229984 or with lower polygenic scores for alcohol consumption drank less than those whose spouses were non‐carriers or had higher polygenic scores (Clarke et al. 2021). Similarly, in the US Health and Retirement Study, individuals whose spouses had higher genome‐wide polygenic predispositions for smoking and drinking tended to smoke and drink more themselves (Otten and Mandemakers 2023). Together, these findings suggest that genetic influences on alcohol‐related behaviors may extend beyond the individual to include the genetic predispositions of social partners.

In the current study, we build on prior work documenting social genetic effects for alcohol outcomes in marital dyads by extending this literature in several important ways. First, whereas prior studies have primarily focused on alcohol consumption, we examine clinically significant alcohol problems as indexed by AUD diagnostic criteria, addressing a gap in the literature. Second, prior studies have largely relied on molecular genetic information (i.e., allelic variation or genome‐wide polygenic scores) to index genetic risk. However, emerging evidence suggests that family history and genome‐wide polygenic scores represent complementary indicators of genetic and environmental liability for substance use disorders and other psychiatric conditions (Dybdahl Krebs et al. 2026; Lai et al. 2022; Mars et al. 2022; Schaefer et al. 2023). Accordingly, we include family history information alongside polygenic scores in our investigation of social genetic effects. Third, spousal similarity in alcohol‐related outcomes may arise through socioeconomic assortment (Mare 1991), whereby individuals select partners with similar socioeconomic factors that are themselves associated with alcohol use behaviors (Huckle et al. 2010; Jones et al. 2015). We therefore examine whether spousal social genetic effects using polygenic scores remain robust after adjusting for both partners' educational attainment. Finally, although the risk associated with spousal social genetic effects may vary as a function of individuals' own genetic predispositions (Mitchell et al. 2013), this possibility has not been formally tested. We address the following questions in a sample of 660 marital dyads of individuals assigned to a genetically inferred European‐like continental group:

1. Do we find evidence for spousal social genetic effects, such that marriage to a spouse with higher genetic predispositions for problematic alcohol use (as indexed with measured and latent indicators including genome‐wide polygenic scores and parental history of alcohol use disorder) is associated with greater AUD clinical criterion counts during marriage?
2. Are spousal social genetic effects explained by socioeconomic assortment (Mare 1991), or do they remain robust after adjusting for both partners' educational attainment?
3. Are spousal social genetic effects stronger among target individuals with higher genetic predispositions for problematic alcohol use?
4. Are there sex differences in social genetic effects?

## 2 Materials and Methods

### 2.1 Participants

Participants came from the Collaborative Study on the Genetics of Alcoholism (COGA) Study, a family‐based study whose objective is to identify genetic and environmental factors implicated in the development of AUD and related psychiatric disorders (Agrawal et al. 2023; Begleiter et al. 1995; Bucholz et al. 2017). Probands were identified through alcohol treatment centers across six sites in the United States. Probands along with their families were invited to participate if the family was sufficiently large (usually sibships greater than 3 with parents available), with two or more family members living in the COGA site catchment area. Comparison families were recruited from the same communities. The institutional review board at all data collection sites approved the study, and written consent was obtained from all participants.

As part of the original sampling strategy, marital dyads were recruited and validated through partners' mutually identified marriage dates from the demographic information collected from participants. In the present study, we restricted the analytic sample to dyads in which both partners: (1) had GWAS data available and were statistically assigned to a genetically inferred European‐like continental group based on similarity to European reference panels, (2) had marital history and AUD information, and (3) had genotypic and phenotypic data needed for the dyadic models. Because actor–partner interdependence models estimate actor and partner effects simultaneously, dyads were included only when both partners had the relevant phenotypic and genotypic data. Analyses were restricted to individuals genetically similar to European reference panels to minimize bias due to population stratification. This resulted in an analytic sample of 1320 individuals in 660 opposite‐sex dyads (age at last assessment: female *M*_age_ = 49.97 years, SD = 12.25; male *M*_age_ = 51.69 years, SD = 12.49). Our analytic sample was limited to legal marriages; cohabiting or marriage‐like relationships were excluded because there was no information collected regarding the timing of relationship formation or dissolution. For participants with multiple marriages, our analyses focused on first marriage for both partners.

### 2.2 Measures

#### 2.2.1 Alcohol Use Disorder Criterion Counts During Marriage (AUDcrit)

Information on the criteria that define lifetime alcohol use disorder according to the Diagnostic and Statistical Manual 5 (DSM‐5; American Psychiatric Association 2013) was obtained from the reliable and valid Semi‐Structured Assessment for the Genetics of Alcoholism (SSAGA) (Bucholz et al. 1994; Hesselbrock et al. 1999). Marital history information was also derived from the SSAGA interview. Because our analyses focused on DSM‐5 AUD criterion counts within the context of marriage, we mapped participants' marital history (age at marriage, age at divorce when applicable) onto criterion‐level onset and recency information to determine whether each criterion was present during marriage. AUD criteria were considered present during marriage if they were active at any point during marriage. Thus, AUD criteria during marriage could reflect persistent problems that began prior to marriage but remained active during marriage, or problems that first developed during marriage. For participants who remained married at their last assessment, AUD criteria were censored at age at last assessment. AUD criteria outside the marital period were set as missing.

#### 2.2.2 Genotyping and Calculating Problematic Alcohol Use Polygenic Scores (PGS_PAU_ )

A full description of genotyping, data processing, quality control, and imputation is available in Lai et al. (2019). Data were imputed to 1000 Genome Phase 3, and single nucleotide polymorphisms (SNPs) with a genotyping rate < 0.95, that violated Hardy–Weinberg equilibrium (*p* < 10^−6^), or had minor allele frequency (MAF) < 0.01 were excluded from analysis.

Genetic similarity principal components were estimated from GWAS data using Eigenstrat (Price et al. 2006) and the 1000 Genomes, Phase 3 reference panel. To reduce confounding from population stratification in analyses of genome‐wide polygenic scores, our analyses were limited to dyads from families statistically assigned to the European‐like continental group based on inferred genetic similarity.

Genetic risk for problematic alcohol use was indexed using genome‐wide polygenic scores for problematic alcohol use (PGS_PAU_). For each individual, PGS_PAU_ was calculated as a weighted sum of effect alleles, with the number of effect alleles at each SNP weighted by the corresponding SNP effect size from GWAS summary statistics. We constructed polygenic scores using PRS‐CS (Ruan et al. 2022). We obtained discovery GWAS summary statistics from a recent meta‐analysis of problematic alcohol use in individuals of European ancestry (Zhou et al. 2023), with the COGA sample removed to avoid overlap between the discovery and target samples. In the COGA European‐like sample, the number of SNPs contributing to PGS_PAU_ was *M* = 1,029,828 (SD = 50,245; range = 841,139–1,098,558), with variation across individuals reflecting genotype availability and overlap with the discovery GWAS summary statistics.

To account for population substructure, we regressed participants' PGS_PAU_ on the first 10 genetic similarity principal components (PC1‐PC10) and saved the residualized PGS. We used standardized, residualized polygenic scores in all downstream analyses.

#### 2.2.3 Parental History of AUD (PH_AUD_ )

Participants were coded as having a parental history of AUD if one or both parents met criteria for AUD based on parent interview using SSAGA. When direct parent interview data were unavailable, we used collateral parental AUD information collected as part of family history reports (Mccutcheon et al. 2018).

#### 2.2.4 Covariates

We included age at last assessment, sex, and relationship length. For individuals who remained married, relationship length was right‐censored at age at last assessment; for those who divorced, relationship length was defined as the number of years between marriage and divorce. Educational attainment was set as the response to the question “What is the highest grade in school you completed.” Responses were converted to the number of years typically required to complete that level of education, ranging from 0 to 17 years: primary or secondary school = actual year; technical school/1 year college = 13 years; 2 years college = 14 years; 3 years college = 15 years; 4 years college = 16 years; any graduate degree = 17 years.

### 2.3 Statistical Analysis

We used actor–partner interdependence models (APIM; Kenny et al. 2006) to test spousal social genetic effects on AUD criterion counts during marriage in spousal dyads. APIM is a multilevel modeling framework that accounts for the interdependence between partners in spousal dyads by estimating actor and partner effects simultaneously within dyads. Figure S1 provides a schematic illustration of this modeling framework using our variables of interest. Actor effects represent associations between an individual's PGS_PAU_ and their own AUDcrit during marriage. Partner effects capture associations between the spouse's PGS_PAU_ and the target individual's AUDcrit, above and beyond the individual's own genetic predispositions. A significant partner effect would provide evidence for social genetic effects. APIM models were estimated using multilevel linear mixed‐effect models with the lme function in R's *nlme* package (Pinheiro et al. 2021), with a dyad‐level random intercept to account for residual non‐independence between partners. The ICCs reported in the tables reflect the within‐dyad correlation captured by the dyad‐level random‐intercept model.

Using this modeling framework, we conducted a series of analyses to examine our research questions. First, we estimated an APIM including PGS_PAU_ at both the actor and partner levels to test whether spousal PGS_PAU_ was associated with the target individual's AUDcrit during marriage. Second, in a subsample of dyads with complete information on PH_AUD_ for both partners (*N* = 165 dyads), we conducted an exploratory APIM including PGS_PAU_ and PH_AUD_ simultaneously as predictors of AUDcrit during marriage. To examine whether spousal social genetic effects indexed by PGS_PAU_ could be explained by socioeconomic assortment, we estimated a model including educational attainment for both partners as predictors of AUDcrit to evaluate whether PGS_PAU_ effects remained robust. We also tested genetic sensitivity to spousal social genetic effects by including a product term between target individual PGS_PAU_ and spousal PGS_PAU_. This model included the lower order main effects of target individual and spousal PGS_PAU_. A statistically significant interaction term would indicate that spousal social genetic effects differ as a function of their own genetic predisposition. Finally, we evaluated potential sex differences in social genetic effects by including sex‐by‐target individual PGS_PAU_ and sex‐by‐spousal PGS_PAU_. This model included the lower order main effects of sex and PGS_PAU_. For the interactive effects models, we did not include additional interactions between other covariates and focal predictors because they were not part of our primary hypotheses and would have increased model complexity and reduced interpretability.

AUDcrit was log‐transformed to adjust for skewness, after adding 1 as a constant because logarithm of zero is undefined. Because AUDcrit was analyzed as a continuous, log‐transformed outcome using linear mixed‐effects models, product terms estimate statistical interaction, or departure from additivity, on the transformed AUDcrit scale (Basten et al. 2025; Knol et al. 2007). We therefore interpret these interactive effects models as tests of moderation on the transformed outcome scale, rather than as multiplicative interactions in an epidemiologic relative‐risk framework. Indices such as the relative excess risk due to interaction (RERI) are designed for relative risk models with binary or time‐to‐event outcomes and were not applicable to the present linear mixed‐effects models. For all a priori hypotheses, we used a *p*‐value threshold of *p* < 0.05 for inference criteria.

### 2.4 Transparency and Openness

The research questions and the analytic plans for the polygenic score analyses were pre‐registered prior to data analysis on the Open Science Framework [osf.io/kdyzn]. Analyses related to parental history of AUD, genetic sensitivity to social genetic effects, and sex differences were developed subsequently and should therefore be considered exploratory. COGA data are available via dbGaP (phs000763.v1.p1, phs000125.v1.p1) or through the National Institute on Alcohol Abuse and Alcoholism.

## 3 Results

### 3.1 Descriptive Statistics and Spousal Correlations

Table 1 presents the descriptives for the key study variables. In this sample, the mean age at marriage was 22.81 years. On average, males had 3.11 AUDcrit during marriage, and females had 0.88 AUDcrit during marriage. Mean years of educational attainment were 13.56 and 13.41 for males and females, respectively. The majority (70.5%) of individuals in the PH_AUD_ subsample had a positive parental history of AUD. Spouses were modestly correlated in AUDcrit during marriage (*r* = 0.14, 95% CI [0.07, 0.22]). Spouses also resembled one another in terms of PGS_PAU_ (*r =* 0.09, 95% CI [0.02, 0.17]) and educational attainment (*r =* 0.48, 95% CI [0.42, 0.54]). Spouses' PH_AUD_ was not correlated (*r =* 0.03, 95% CI [−0.12, 0.18]).

**TABLE 1:** Descriptive statistics for key study variables for spousal dyads (by sex).

	Males	Females
	*M* (SD)/%	*M* (SD)/%
Age at last assessment	51.69 (12.49)	49.97 (12.25)
Age at marriage	23.74 (3.79)	21.88 (3.37)
Age at divorce	38.88 (10.59)	37.44 (10.57)
Length of marriage (with divorce)	15.80 (9.78)	16.37 (9.82)
Educational attainment (years)	13.56 (2.43)	13.41 (2.18)
Parental history of AUD	73.8%	67.2%
AUDcrit during marriage	3.11 (3.72)	0.88 (2.14)

*Note:* Mean and standard deviation are provided for continuous measure, and percentage is provided for dichotomous measure.

Abbreviation: AUDcrit, alcohol use disorder criterion counts during marriage.

### 3.2 Do Spouse Genetic Predispositions Predict Target Individual AUDcrit During Marriage?

#### 3.2.1 Model with PGS_PAU_

We first tested whether the target individual's and spouse polygenic predispositions for problematic alcohol use were associated with the target individual's AUDcrit (Table 2). Consistent with hypotheses, target individuals with higher PGS_PAU_ had more AUDcrit during marriage, indicating an actor effect. In addition, target individuals had more AUDcrit during marriage when their partner had a higher PGS_PAU_, demonstrating a partner effect above and beyond the target individual's own polygenic predispositions (B = 0.084, 95% CI [0.035, 0.132]). Because PGS_PAU_ was standardized and AUDcrit was log‐transformed after adding 1, this estimate indicates that a 1‐SD higher spousal PGS_PAU_ was associated with a 0.084‐unit higher log‐transformed AUDcrit during marriage, equivalent to approximately 8.8% higher AUDcrit on the transformed scale.

**TABLE 2:** Multilevel analysis predicting alcohol use disorder criterion counts during marriage as a function of target individual and spousal problematic alcohol use polygenic scores.

Parameter	*B*	95% CI	*p*
Target individual age	**−0.224**	**[−0.331, −0.117]**	**< 0.0001**
Target individual sex (1 = male)	**0.407**	**[0.361, 0.453]**	**< 0.0001**
Target individual relationship length	**0.175**	**[0.068, 0.281]**	**0.0014**
Target individual PGS_PAU_	**0.200**	**[0.152, 0.248]**	**< 0.0001**
Spousal PGS_PAU_	**0.084**	**[0.035, 0.132]**	**0.0007**
Random effects			
Random intercept variance (DYAD_ID)	0.105		
Residual variance	0.691		
Intraclass correlation (ICC)	0.132		

*Note:* PGS residualized on the first 10 genetic similarity principal components. Relationship length was computed using age at divorce or, for non‐divorced couples, age at last assessment and censored accordingly. Significant results are indicated by boldface (*p* < 0.05).

Abbreviations: CI, confidence interval; PGS_PAU_, problematic alcohol use polygenic score.

#### 3.2.2 Model with PGS_PAU_ and PH_AUD_

We next examined whether target individual and spousal genetic predispositions, indexed by PGS_PAU_ and PH_AUD_, predicted AUDcrit during marriage in the subsample of dyads with complete PGS_PAU_ and PH_AUD_ data for both partners (*n* = 330 individuals nested in 165 dyads; Table 3). In this model, target individuals with higher PGS_PAU_ and those with a positive parental history of AUD had higher AUDcrit during marriage, indicating unique actor effects. There were no statistically significant partner effects, although the estimate for spousal PGS_PAU_ remained in the same direction and of similar magnitude as in the prior model.

**TABLE 3:** Multilevel analysis predicting alcohol use disorder criterion counts during marriage as a function of target individual and spousal problematic alcohol use polygenic scores and parental history of alcohol use disorder.

Parameter	*B*	95% CI	*p*
Target individual age	−0.103	[−0.272, 0.067]	0.2338
Target individual sex (1 = male)	**0.464**	**[0.375, 0.553]**	**< 0.0001**
Target individual relationship length	−0.070	[−0.238, 0.098]	0.4137
Target individual PGS_PAU_	**0.093**	**[0.000, 0.187]**	**0.0494**
Target individual PH_AUD_	**0.172**	**[0.079, 0.266]**	**0.0004**
Spousal PGS_PAU_	0.080	[−0.013, 0.173]	0.0917
Spousal PH_AUD_	0.019	[−0.074, 0.112]	0.6876
Random effects			
Random intercept variance (DYAD_ID)	0.081		
Residual variance	0.634		
Intraclass correlation (ICC)	0.113		

*Note:* PGS residualized on the first 10 genetic similarity principal components. Relationship length was computed using age at divorce or, for non‐divorced couples, age at last assessment and censored accordingly. Significant results are indicated by boldface (*p* < 0.05).

Abbreviations: CI, confidence interval; PGS_PAU_, problematic alcohol use polygenic score; PH_AUD_, parental history of alcohol use disorder.

### 3.3 Model Adjusting for Target Individual and Spousal Educational Attainment

To evaluate the robustness of social genetic effects of PGS_PAU_, we conducted post hoc sensitivity analyses adjusting for both partners' educational attainment (Table 4). The pattern of associations was largely consistent with results from the PGS_PAU_ models. Target individuals had higher AUDcrit when they had a higher PGS_PAU_, and importantly, individuals also had higher AUDcrit when their spouse had a higher PGS_PAU_, above and beyond the effects of their own PGS_PAU_ and both partners' educational attainment. Higher target individual educational attainment was associated with lower AUDcrit, whereas spousal educational attainment was not significantly associated with target individual's AUDcrit.

**TABLE 4:** Multilevel analysis predicting alcohol use disorder criterion counts during marriage as a function of target individual and spousal problematic alcohol use polygenic scores, adjusting for both partners' educational attainment.

Parameter	*B*	95% CI	*p*
Target individual age	**−0.196**	**[−0.301, −0.091]**	**0.0003**
Target individual sex (1 = male)	**0.408**	**[0.363, 0.454]**	**< 0.0001**
Target individual relationship length	**0.132**	**[0.027, 0.237]**	**0**.**0137**
Target individual PGS_PAU_	**0.187**	**[0.140, 0.235]**	**< 0.0001**
Spousal PGS_PAU_	**0.073**	**[0.026, 0.121]**	**0.0026**
Target individual educational attainment	**−0.146**	**[−0.200, −0.093]**	**< 0.0001**
Spouse educational attainment	−0.029	[−0.082, 0.024]	0.2791
Random effects			
Random intercept variance (DYAD_ID)	0.086		
Residual variance	0.684		
Intraclass correlation (ICC)	0.112		

*Note:* PGS residualized on the first 10 genetic similarity principal components. Relationship length was computed using age at divorce or, for non‐divorced couples, age at last assessment and censored accordingly. Significant results are indicated by boldface (*p* < 0.05).

Abbreviations: CI, confidence interval; PGS_PAU_, problematic alcohol use polygenic score.

### 3.4 Are Spousal Social Genetic Effects Stronger Among Target Individuals With Higher PGS_PAU_ ?

To test whether spousal social genetic effects varied as a function of target individuals' own genetic predispositions, we included an interaction term between target individual PGS_PAU_ and spousal PGS_PAU_ (Table 5). The interaction term was not statistically significant, providing no evidence that the spousal PGS_PAU_ effect was stronger among target individuals with higher PGS_PAU_.

**TABLE 5:** Multilevel analysis predicting alcohol use disorder criterion counts during marriage as a function of target individual and spousal problematic alcohol use polygenic scores and two‐way interaction terms between target individual and spousal polygenic scores.

Parameter	*B*	95% CI	*p*
Target individual age	**−0.224**	**[−0.332, −0.117]**	**< 0.0001**
Target individual sex (1 = male)	**0.407**	**[0.361, 0.453]**	**< 0.0001**
Target individual relationship length	**0.175**	**[0.068, 0.281]**	**0.0014**
Target individual PGS_PAU_	**0.200**	**[0.152, 0.249]**	**< 0.0001**
Spousal PGS_PAU_	**0.084**	**[0.036, 0.132]**	**0.0007**
Target individual PGS_PAU_ × Spousal PGS_PAU_	−0.010	[−0.061, 0.042]	0.7151
Random effects			
Random intercept variance (DYAD_ID)	0.105		
Residual variance	0.691		
Intraclass correlation (ICC)	0.132		

*Note:* PGS residualized on the first 10 genetic similarity principal components. Relationship length was computed using age at divorce or, for non‐divorced couples, age at last assessment and censored accordingly. Significant results are indicated by boldface (*p* < 0.05).

Abbreviations: CI, confidence interval; PGS_PAU_, problematic alcohol use polygenic score.

### 3.5 Sex Differences in Social Genetic Effects

To test whether social genetic effects differed by sex, we included an interaction term between the target individual's sex and spousal PGS_PAU_ (Table 6). This interaction was statistically significant, indicating that the association between spousal PGS_PAU_ and target individual's AUDcrit during marriage differed for males and females. The association between spousal PGS_PAU_ and target individual AUDcrit was stronger and statistically significant among male target individuals (*b* = 0.14, *p* < 0.01), whereas it was not significant among female target individuals (*b* = 0.02, ns). In other words, having a spouse with higher PGS_PAU_ was independently associated with higher AUDcrit during marriage among male target individuals, but not among female target individuals.

**TABLE 6:** Multilevel analysis predicting alcohol use disorder criterion counts during marriage as a function of target individual and spousal problematic alcohol use polygenic scores and two‐way interaction terms between sex and polygenic scores.

Parameter	*B*	95% CI	*p*
Target individual age	**−0.225**	**[−0.332, −0.118]**	**< 0.0001**
Target individual sex (1 = male)	**0.815**	**[0.723, 0.906]**	**< 0.0001**
Target individual relationship length	**0.175**	**[0.068, 0.281]**	**0.0014**
Target individual PGS_PAU_	**0.179**	**[0.112, 0.247]**	**< 0.0001**
Spousal PGS_PAU_	0.017	[−0.052, 0.086]	0.6286
Target individual sex × Target individual PGS_PAU_	0.041	[−0.056, 0.138]	0.4040
Target individual sex × Spousal PGS_PAU_	**0.130**	**[0.034, 0.227]**	**0.0083**
Random effects			
Random intercept variance (DYAD_ID)	0.107		
Residual variance	0.684		
Intraclass correlation (ICC)	0.135		

*Note:* PGS residualized on the first 10 genetic similarity principal components. Relationship length was computed using age at divorce or, for non‐divorced couples, age at last assessment and censored accordingly. Significant results are indicated by boldface (*p* < 0.05).

Abbreviations: CI, confidence interval; PGS_PAU_, problematic alcohol use polygenic score.

### 3.6 Sensitivity Analyses

Because proband status was associated with sex in the analytic sample of dyads, we conducted a sensitivity analysis adjusting for both target individual and spousal proband status (Table 7). Among the 176 individuals identified as probands, 123 (69.9%) were male and 53 (30.1%) were female. However, most male partners in the analytic sample were not probands, with 123 of 660 male partners (18.6%) identified as probands. The overall pattern of findings was substantively unchanged with spousal PGS_PAU_ remained significantly associated with higher AUDcrit during marriage (*B* = 0.091, 95% CI [0.044, 0.137]) after accounting for target individual PGS_PAU_.

**TABLE 7:** Multilevel analysis predicting alcohol use disorder criterion counts during marriage as a function of target individual and spousal problematic alcohol use polygenic scores, adjusting for both partners' proband status.

Parameter	*B*	95% CI	*p*
Target individual age	**−0.239**	**[−0.344, −0.134]**	**< 0.0001**
Target individual sex (1 = male)	**0.371**	**[0.326, 0.416]**	**< 0.0001**
Target individual relationship length	**0.200**	**[0.095, 0.305]**	**0.0002**
Target individual PGS_PAU_	**0.186**	**[0.139, 0.233]**	**< 0.0001**
Spousal PGS_PAU_	**0.091**	**[0.044, 0.137]**	**0.0002**
Target individual proband status	**0.636**	**[0.492, 0.779]**	**< 0.0001**
Spousal proband status	−0.069	[−0.213, 0.075]	0.3453
Random effects			
Random intercept variance (DYAD_ID)	0.122		
Residual variance	0.627		
Intraclass correlation (ICC)	0.163		

*Note:* PGS residualized on the first 10 genetic similarity principal components. Relationship length was computed using age at divorce or, for non‐divorced couples, age at last assessment and censored accordingly. Significant results are indicated by boldface (*p* < 0.05).

Abbreviations: CI, confidence interval; PGS_PAU_, problematic alcohol use polygenic score.

### 3.7 Exploratory Analysis in the African‐like Dyads

Although the primary analyses focused on genetically inferred European‐like dyads to minimize population stratification and maximize statistical power, COGA also includes a subsample of participants genetically similar to African reference panels. We therefore conducted parallel analyses in the smaller African‐like dyad sample (*n* = 136) to evaluate whether a similar pattern of findings was observed in the African‐like group. In this exploratory analysis (Table 8), target individual PGS_PAU_ was associated with higher AUDcrit (*B* = 0.199, 95% CI [0.034, 0.365], *p* = 0.018), whereas spousal PGS_PAU_ was not associated with target individual's AUDcrit at a statistically significant level (*B* = −0.027, 95% CI [−0.193, 0.138], *p* = 0.742).

**TABLE 8:** Multilevel analysis predicting alcohol use disorder criterion counts during marriage as a function of target individual and spousal problematic alcohol use polygenic scores among African‐like individuals.

Parameter	*B*	95% CI	*p*
Target individual age	−0.144	[−0.182, 0.167]	0.3661
Target individual sex (1 = female)	**−0.412**	**[−0.46, 0.172]**	**< 0.001**
Target individual relationship length	**0.328**	**[−0.536, −0.289]**	**0.** **0410**
Target individual PGS_PAU_	**0.199**	**[0.014, 0.642]**	0.**0188**
Spousal PGS_PAU_	−0.027	[0.034, 0.365]	0.742
Random effects			
Random intercept variance (DYAD_ID)	0.271		
Residual variance	0.496		
Intraclass correlation (ICC)	0.353		

*Note:* PGS residualized on the first 10 genetic similarity principal components. Relationship length was computed using age at divorce or, for non‐divorced couples, age at last assessment and censored accordingly. Significant results are indicated by boldface (*p* < 0.05).

Abbreviations: CI, confidence interval; PGS_PAU_, problematic alcohol use polygenic score.

## 4 Discussion

We used dyadic genetic and clinical information to investigate social genetic effects for AUD within marriage in a high‐risk sample. We indexed partners' genetic predispositions in two ways. The first was a genome‐wide polygenic score for problematic alcohol use, derived from GWAS summary statistics on a sample of more than 1 M individuals (Zhou et al. 2023). The second index was a parental history of AUD, based largely on direct psychiatric interviews with clinical diagnostic information, although these data were available for only a limited proportion of our overall sample. Consistent with our hypothesis, spousal polygenic predispositions were associated with the target individual's AUD criterion counts during marriage. This social genetic effect remained statistically significant even after adjusting for both partners' educational attainment. These findings are consistent with prior reports regarding spousal social genetic influences on alcohol use behaviors (Clarke et al. 2021; Otten and Mandemakers 2023) and add to a growing body of evidence indicating that characteristics of spouses, including their genetic makeup, can influence health‐related outcomes.

Importantly, our dyadic modeling approach accounted for both direct genetic effects and the correlations between spouses' genetic predispositions, as well as residual correlation in their clinically significant alcohol problems during marriage. Direct genetic effects, or the association between an individual's own genetic predispositions and AUD criterion count during marriage, were approximately twice the magnitude of the spousal genetic effects as measured with polygenic scores. The relative magnitude of direct versus social genetic effects observed in COGA largely mirrors prior reports of drinks per week (Otten and Mandemakers 2023), alcohol consumption (Clarke et al. 2021), and AUD (Salvatore et al. 2020). Spouses also modestly resembled one another in their genetic predispositions for problematic alcohol use, as well as in the number of AUD criterion counts during marriage. Spousal resemblance for PGS_PAU_ in our sample was higher than spousal resemblance for a drinks per week polygenic score reported by others (Otten and Mandemakers 2023). Several study differences may help explain this pattern. First, the polygenic scores used in the two studies was based on different discovery GWAS phenotypes. Because the genetic factors associated with alcohol consumption only partially overlap with the genetic factors associated with alcohol problems and alcohol use disorders (Sanchez‐Roige et al. 2020), these scores may capture partially overlapping but non‐identical dimensions of alcohol liability. Second, COGA is a high‐risk sample with participants from extended families enriched for AUD. This may have contributed to higher spousal resemblance to the extent that individuals with greater alcohol‐related genetic predispositions are more likely to partner with others who share similar liabilities or background characteristics. Finally, differences in sample characteristics, including the older age of the Health and Retirement Study sample used by Otten and Mandemakers (2023), may also contribute to differences across studies. We note, however, that the magnitude of spousal resemblance for AUD observed here was largely consistent with other reports from studies of assortative mating. For example, spousal resemblance (measured with tetrachoric correlations) for DSM‐III‐R lifetime alcohol use disorder diagnoses was r_tet_ = 0.12 and 0.07 in two population‐based samples (Maes et al. 1998).

We did not find support for our hypothesis that a spouse's parental history of AUD was associated with AUDcrit. This contrasts with a previous report from our group using Swedish national registry data, in which spousal parental history of AUD was associated with likelihood of AUD registration during marriage (Salvatore et al. 2020). The null association observed here may reflect lower statistical power, as analyses involving PH_AUD_ were limited to a smaller number of dyads with complete PH_AUD_ information. As with any null result, however, we caution against interpreting this as evidence that spousal PH_AUD_ is not associated with AUDcrit. It is also noteworthy that the majority (> 70%) of participants in our sample with available parental history data had an AUD‐affected parent. This pattern is consistent with the high‐risk nature of the COGA sample, and the relatively limited variation in PH_AUD_ may have further limited our ability to detect statistically significant effects. Future studies of social genetic effects that incorporate both family history information and molecular genetic information of liability may help clarify whether and how these complementary measures of genetic risk are related to social partners' alcohol‐related and broader behavioral outcomes.

When we explored sex differences in social genetic effects, we found evidence that the influence of a spouse's genetic predispositions on target individual's AUD criteria during marriage was stronger for male than female target individuals. This pattern is consistent with phenotypic research suggesting that, in opposite‐sex marriages, males' health behaviors are often more strongly shaped by their spouses than the reverse (Umberson 1992). Although the emerging literature on social genetic effects has generally reported limited evidence for sex differences, including analyses of spousal dyads from the Health and Retirement Study that found no sex‐specific social genetic effects for drinking or smoking (Otten and Mandemakers 2023), our findings suggest that such differences may emerge in the context of clinically significant alcohol problems. One possible explanation is that social norms within opposite‐sex partnerships often place females in roles that shape day‐to‐day household routines (Umberson et al. 2018), health‐related behaviors (Markey et al. 2008; Umberson et al. 2018), and emotional regulation (Duncombe and Marsden 1993) within relationships. As male partners' drinking progresses to clinically significant alcohol problems, these relational dynamics may become particularly relevant. In this context, genetically influenced characteristics of female spouses may shape shared routines, health‐related behaviors, and emotional contexts that influence male partners' AUD‐related outcomes. However, the mechanisms underlying these sex‐specific social genetic effects remain unclear and warrant further investigation.

We also examined whether individuals' own genetic predispositions moderated social genetic effects. Contrary to our expectation, we found no evidence that the social genetic effect was stronger among target individuals with higher polygenic predispositions for problematic alcohol use. This finding is inconsistent with the results in our earlier report examining social genetic effects among adolescent peers, in which individuals at higher genetic risk appeared more sensitive to the pathogenic effects of peers' genetic predispositions for AUD (Salvatore et al. 2024). However, we note that genetic predispositions in that study were indexed using an inferred measure (i.e., pedigree‐based family history) rather than with polygenic scores.

## 5 Implications

Establishing the plausibility of social genetic effects for AUD has important implications for understanding the complex pathways linking genetic variation to the experience of clinically significant problems with alcohol. Our findings add to a growing body of evidence suggesting that the genetic makeup of social partners may be associated with individuals' behavioral health outcomes beyond spousal phenotypic resemblance that may be attributable to assortative mating or shared environments. Importantly, both polygenic scores and parental history of AUD may reflect vulnerability to problematic alcohol use as well as to a broader range of genetically correlated traits and behaviors, including impulsivity and depression (Slutske et al. 2002; Zhou et al. 2023). These traits are known to pose challenges to romantic relationship functioning, satisfaction, and stability (Joel et al. 2020; Righetti and Finkenauer 2011) and may represent potential pathways through which partners' genetic predispositions are associated with alcohol‐related problems. Further work is needed to clarify the mechanisms through which these associations arise within marriage and to identify opportunities to disrupt the social transmission of risk between spouses.

## 6 Limitations

Although our study has several strengths including a large dyadic sample, measures of both polygenic indices and parental history of AUD, and gold standard clinical diagnostic information, these results should be considered in the context of several limitations. First, the COGA sample is enriched with individuals with alcohol and other substance use disorders; accordingly, the results may not generalize to other populations. Second, although COGA is a diverse sample, our primary analyses focused on individuals genetically similar to European genomic reference panels, which was the largest genetic similarity group with complete dyadic data and because polygenic score performance differs across populations (Martin et al. 2017). Our exploratory analyses in the much smaller African‐like dyad sample showed evidence of direct genetic effects but not a spousal PGS_PAU_ effect. However, these analyses were underpowered for detecting partner effects and should be interpreted cautiously. Larger dyadic samples inclusive of multiple populations, together with continued development of polygenic scoring methods across populations, will be needed to evaluate the generalizability of spousal social genetic effects across groups.

Third, the analytic sample was limited to legally married couples because information on the timing of relationship formation and dissolution was collected only for legal marriages; comparable data were not available for cohabiting or other long‐term partnerships. Fourth, our polygenic indices of genetic predispositions only capture common genetic variation. Fifth, we recognize that both molecular and latent indicators of genetic predispositions may also reflect rearing environmental effects and therefore do not represent decontextualized measures of genetic liability. Disentangling social genetic effects from social rearing environmental effects would require study designs in which these influences can be separated, such as in dyads where one spouse was adopted (Salvatore et al. 2020). Finally, although many of our effects were statistically significant, the effect sizes were small.

## 7 Conclusions

In a high‐risk sample, marriage to a spouse with a greater genetic predisposition for problematic alcohol use was associated with higher DSM‐5 AUD criterion counts during marriage. These results highlight social genetic effect pathways as an important dimension of AUD risk and underscore that an individual's genetic predispositions for problematic alcohol use may exert influences that extend “beyond the skin.”

## Funding

This work was supported by the National Institutes of Health (NIH) Grant R01AA030996 (PI: Salvatore) from the National Institute on Alcohol Abuse and Alcoholism (NIAAA). The Collaborative Study on the Genetics of Alcoholism (COGA) is supported by NIH Grant U10AA008401 (PI: Porjesz). Dongbing Lai is supported by R01AA031176 from NIAAA.

## Conflicts of Interest

Dr. Danielle Dick is a Co‐founder and Chief Scientific Officer for Thrive Genetics Inc. She is on the Advisory Board for the Seek Women's Health Company. She has received royalties from Penguin Random House for her book, *The Child Code: Understanding Your Child's Unique Nature for Happier, More Effective Parenting*. All other authors declare no potential conflict of interest.

## Supporting information


**Figure S1:** acer70399‐sup‐0001‐FigureS1.docx.

## Data Availability

COGA data are available via dbGaP (phs000763.v1.p1, phs000125.v1.p1) or through the National Institute on Alcohol Abuse and Alcoholism.
