# Supplementary material for: The Influence of Spousal Genotype on Alcohol Problems in Marriage
Source: Alcohol Clin Exp Res (Hoboken). 2026 Aug 20;50(8):e70399. doi: 10.1111/acer.70399 (PMC13494056; doi:10.1111/acer.70399)
Supplement: Supplementary file 1 [file ACER-50-0-s001.docx]

Supplementary Materials for

“**The Influence of Spousal Genotype on Alcohol Problems in Marriage”**

**Supplemental Figure S1**

Schematic illustration of a multilevel Actor-Partner Interdependence Model (APIM), depicting variables of interest in the current study. Paths labeled *a* denote actor effects, and paths labeled *p* denote partner effects. The curved double-headed arrow between residuals represents residual non-independence between partners’ AUD criterion counts during marriage. In our multilevel implementation of the APIM, this non-independence was modeled using a dyad-level random intercept.
